# A cohort study of the impact of COVID‐19 on the quality of life of people newly diagnosed with dementia and their family carers

**DOI:** 10.1002/trc2.12236

**Published:** 2022-05-02

**Authors:** Ben Hicks, Sanna Read, Bo Hu, Raphael Wittenberg, Amanda Grahamslaw, Anomita Karim, Evelyn Martin, Eleanor Nuzum, Jacob Reichental, Alice Russell, Elaine Siddle, Bryony Storey, Eva Tipping, Kate Baxter, Yvonne Birks, Carol Brayne, Nicola Brimblecombe, Margaret Dangoor, Josie Dixon, Kate Gridley, Peter R. Harris, Martin Knapp, Eleanor Miles, Rotem Perach, Louise Robinson, Jennifer Rusted, Rob Stewart, Alan J. Thomas, Sube Banerjee

**Affiliations:** ^1^ Brighton and Sussex Medical School University of Sussex Brighton UK; ^2^ Care Policy and Evaluation Centre London School of Economics and Political Science London UK; ^3^ Gateshead Health NHS Foundation Trust Gateshead UK; ^4^ Sussex Partnership NHS Foundation Trust Worthing UK; ^5^ South London and Maudsley NHS Foundation Trust London UK; ^6^ Social Policy Research Unit University of York York UK; ^7^ Cambridge Public Health University of Cambridge Cambridge UK; ^8^ School of Psychology University of Sussex Brighton UK; ^9^ Population Health Sciences Institute Newcastle University Newcastle UK; ^10^ Institute of Psychiatry Psychology and Neuroscience King's College London London UK; ^11^ Institute for Ageing Newcastle University Newcastle UK; ^12^ Faculty of Health University of Plymouth Plymouth UK

**Keywords:** cohort study, COVID‐19, dementia, inequalities, inequities, post‐diagnostic care, quality of life

## Abstract

**Introduction:**

COVID‐19 has impacted people with dementia and their family carers, yet little is known about effects on overall quality of life.

**Methods:**

In a UK cohort study, pre‐ and post‐pandemic data were collected from 114 carers and 93 recently diagnosed people with dementia. Latent growth curve modeling examined change in quality of life.

**Results:**

Carers reported significant decline in quality of life, although no change was demonstrated by people with dementia. In multivariable analyses, higher levels of cognitive impairment, deprivation, study site, and lower number of memory clinic contacts were associated with greater decline in carer quality of life.

**Discussion:**

Maintaining life quality for people with dementia during the pandemic appears to have come at the expense of their family carers. This inequity has fallen hardest on those caring for people with more severe dementia, in deprived areas, and with least support from memory services. These effects may be prevented or reversed by post‐diagnostic care.

## 1 INTRODUCTION

Quality of life (QoL) is a complex, multidimensional construct that is widely used to examine people's experiences of living with dementia.^1^, ^2^ The World Health Organization defines it as the “evaluation by an individual of their position in life, assessed in context of one's culture, values, goals, expectations, standards, and concerns” (p. 1405).^3^ Research has highlighted a range of modifiable components that differ for people with dementia^2^, ^4^, ^5^ and family carers,^1^, ^6^ and can enhance or hinder their QoL. These include demographic (e.g., spouse/non‐spouse carer status and living situation), personal (e.g., coping strategies), social (e.g., familial networks), and contextual factors (e.g., ability to contribute to their community). Consequently, anything that may impede the access to, and quality of, these resources is likely to result in a worsening of QoL for these populations.

In late 2019, a new coronavirus (COVID‐19) emerged causing global disruption as countries introduced public health strategies to attempt to control the spread of infection. Since March 2020, the UK has imposed a series of measures including national and regional lockdowns, prolonged periods of physical/social distancing, reduced access to local services and community facilities, “shielding” for vulnerable groups, and travel restrictions. The pandemic has had particular negative effects on people with dementia and their carers.^7^, ^8^, ^9^ People with dementia are at high risk of infection and death,^10^, ^11^ accounting for 25% to 31% of all UK COVID‐19–related deaths.^12^ In the United States people with dementia were at significantly increased risk for COVID‐19, and this was particularly the case for Black people and those with vascular dementia.^13^ This is driven by the association of dementia with age and biological vulnerability, and the social vulnerability of the high prevalence of people with dementia living in care homes where mortality from COVID‐19 has been particularly high, exacerbated in the UK by the practice of discharging infected individuals from hospitals to care homes.^14^ People with dementia also have difficulties in remembering and understanding restrictions and precautions^15^, ^16^ and may find government guidance inaccessible and difficult to follow, particularly without the support of a carer.^17^

COVID‐19 governmental public health strategies have restricted socialization and reduced access to health and social care services and community activities, potentially affecting mental health and well‐being.^18^, ^19^ Researchers have described the “shrinking worlds” of some people with dementia during the pandemic, finding themselves unable to engage in meaningful activities and social interaction that had previously provided a sense of purpose, identity, and social connectedness.^17^ However, they also noted that for some the “lockdown bubble” provided a break from the busyness of the outside world, where they could feel safer. Through the pandemic carers in general (i.e., not just those caring for people with dementia) have reported higher levels of depressive symptoms and anxiety than non‐carers^20^ and increasing levels of depression.^21^ Researchers, examining the impact of social support service closures on the mental well‐being of a combined sample of older people, people with dementia, and carers during the first 3 months of the pandemic, found levels of self‐reported anxiety dropped through this period, but depression rose. Self‐reported QoL (measured by Warwick‐Edinburgh Mental Wellbeing Scale, which is not dementia‐specific) increased for people with dementia and older adults.^22^

These early studies paint a mixed picture of the impact of the pandemic and subsequent restrictions on the QoL of people with dementia and their carers. To date, most studies have been qualitative or used cross‐sectional surveys, relying on subjective retrospective ratings, with few studies comparing findings in the pandemic to pre‐pandemic measures.^23^ One small Spanish study compared carer‐reported neuropsychiatric symptoms of 20 people with mild Alzheimer's disease (AD) using data collected 1 month before lockdown and then re‐evaluating them after 5 weeks of lockdown.^24^ The findings showed increases in apathy, agitation, and aberrant motor behavior for their participants with dementia, although no significant decreases were reported in well‐being (as measured by carer proxy reports on the EQ‐5D). This study (DETERMIND‐C19) aimed to address this evidence gap by examining QoL in a cohort of people newly diagnosed with dementia who were recruited into the DETERMIND program in the months before the first COVID‐19 lockdown.^25^ The cohort was comprehensively assessed prior to the pandemic, providing the opportunity to conduct an in‐pandemic assessment allowing for the investigation of how participants were affected. We hypothesized that the impact of the COVID‐19 pandemic would be to decrease QoL for people with dementia and their family carers.

## 2 METHODS

### 2.1 Study design

DETERMIND‐C19 used a mixed methods design involving quantitative interviews with people with dementia and carers recruited into the DETERMIND program, followed by in‐depth qualitative interviews with a subsample. This article presents the findings of the quantitative element. DETERMIND recruited a cohort of 900 people with newly diagnosed dementia and their carers in three areas of England (North‐East, South London, and Sussex).^25^ DETERMIND is inclusive of all participants with a clinical diagnosis of any dementia (rather than mild cognitive impairment or subjective memory complaints) that have been diagnosed within 6 months of baseline assessment. Baseline interviews started in late July 2019 and were paused in March 2020 because of the pandemic. By then 261 people with dementia and 206 carers had been interviewed. The DETERMIND‐C19 study reported here examines the impact of the COVID‐19 pandemic on this cohort. While all carers were eligible to participate, only those people with dementia who had the capacity to provide informed consent at DETERMIND baseline were approached because of the difficulty of assessing capacity over the telephone. Ethics approval for the DETERMIND and DETERMIND‐C19 studies were obtained by the Health Research Authority Brighton and Sussex Research Ethics Committee (REC 19/LO/0528. IRAS 261263).

### 2.2 Procedure

Baseline DETERMIND interviews were completed face‐to‐face and follow‐up DETERMIND‐C19 interviews were conducted between July and October 2020 by telephone (because face‐to‐face visits were not permitted due to government restrictions) with people with dementia and/or their carers. Data were collected on topics including: QoL, physical and mental health, service use, social connections, and perception of the pandemic.

**RESEARCH IN CONTEXT**

1. **Systematic review**: People with dementia are at high risk of adverse outcomes from COVID‐19 and governmental control measures. Reductions in meaningful activity and social interaction have resulted in depression but the “lockdown” may have provided some a break and sense of security. The literature has limitations, using retrospective ratings collected during the pandemic rather than comparing findings in the pandemic to pre‐pandemic data.
2. **Interpretation**: Overall people with dementia's quality of life (QoL) has stayed steady during the pandemic, but possibly at the expense of family carer QoL, particularly those caring for people with more severe dementia and living in more deprived areas. Negative effects might be mitigated by post‐diagnostic care.
3. **Future directions**: The evidence of resilience of people with dementia suggests future research should examine positive as well as negative outcomes. Attention is needed to address inequities for those caring for people with more severe dementia and those living in more deprived areas.

Prior to all telephone interviews, participants were sent prompt cards, outlining response scales for each of the questionnaires, and encouraged to use them during data collection. It was envisaged that this would ameliorate some of the challenges associated with working memory that may be encountered by people with dementia and so enhance the validity of the data obtained.

### 2.3 Measures

We measured self‐rated and carer‐rated QoL of people with dementia using the 28‐item Dementia Quality of Life Instrument (DEMQOL; range 28–112) and 31‐item DEMQOL‐Proxy (range 31–124) scales.^26^ These are interviewer‐administered, dementia‐specific questionnaires for assessing health‐related QoL and include items focused on emotions, memory, and activities of daily life in the past week. The DEMQOL‐Proxy elicits carer's perceptions of QoL for the person with dementia, with their answering from the viewpoint of the person with dementia rather than themselves. It was used to collect data from all people with dementia who had a carer participating in the study. To assess carer QoL we used C‐DEMQOL (range 30–150), which has 30 items.^27^ This is an interviewer‐administered, dementia‐specific questionnaire that captures how people have felt in their caring role over the past 4 weeks. It includes items concerned with the carer's responsibilities and needs, well‐being, role, support, and feelings about the future. In all these instruments higher scores represent higher QoL.

Research has demonstrated that a range of demographic determinants may influence QoL for people with dementia and/or their carers, alongside dementia characteristics and contextual resources such as access and use of services.^1^, ^2^ Consequently, these variables were collected and incorporated within our analysis. These included: study site, co‐residence of the person with dementia and the carer, age at baseline, sex, ethnicity, marital status, education, occupational class based on the National Statistics Socio‐economic Classification (NS‐SEC),^28^ work status, home ownership, Office of National Statistics (ONS) rural–urban classification of the post code area,^29^ deciles of Index of Multiple Deprivation (IMD) based on post codes,^30^ and the number of months between the baseline and C19 interview. Some information was collected only for the person with dementia including receipt of social security benefits (Pension Credit and Attendance Allowance or Disability Living Allowance). Severity of cognitive impairment was measured using the Mini‐Mental State Examination (MMSE).^31^ Type of dementia (AD, vascular, Lewy body, mixed, and other) and the number of months since the diagnosis of dementia was given were also included. Number of hours per day the carer provided care for the person with dementia was used to measure intensity of caregiving. Respondents reported the number of memory clinic contacts in the 3 months before the baseline assessment (DETERMIND) and the 3 months before the DETERMIND‐C19 interview.

### 2.4 Analysis

Latent growth curve modeling examined the level and change in QoL and its associations with characteristics of the carer and person with dementia. In a latent growth curve model,^32^ random effects are used to capture individual differences and fixed effects to estimate the average growth of the entire sample. Analyses were carried out with Mplus 8.^33^ As there were only two time points, a simple linear change (slope) in QoL could be estimated with the initial level (intercept). Participant characteristics collected at baseline were included as potential predictors of the level and change in QoL. As there was very little change in the characteristics between the baseline and C19 interview, the baseline value was used. MMSE was only available at baseline. Number of hours of care showed some individual change, but the sensitivity analysis using it time‐varying suggested that the baseline value was a stronger predictor of QoL in the C19 interview than the concurrent one. Therefore, we used only the baseline number of hours of care. Memory clinic contacts were used as a time‐varying predictor. The sample size was too small to include all characteristics in the same model. Therefore, we fitted the models first for each type of characteristic separately (bivariate models) to identify those associated with the level or change in QoL. We then included the bivariate predictors that were associated with either interact or slope of QoL in a multivariable model. All multivariable models were adjusted for age, sex, and the number of months between the baseline and C19 interview. The fit of the model was assessed by Chi‐square analysis (a *P*‐value > .05 recommended as a good fit), but as this is sensitive to sample size (Chi‐square *P*‐value < .05 when samples sizes reach 200+^34^), we used three other recommended fit indices:^35^ the comparative fit index (CFI), root mean square error of approximation (RMSEA), and standardized root mean square residual (SRMR). A value at or below 0.05 for the RMSEA and SRMR and at or above 0.95 for the CFI indicated a good fit for the model. Maximum likelihood estimation with robust standard errors (MLR) was used to take into account any sample non‐normality. Missing data were handled using the full information maximum likelihood method (FIML),^36^ which makes it possible to include cases with missing values for any dependent variable in path models such that information on the means and variances of all data are used.

## 3 RESULTS

### 3.1 Participants

Interviews were conducted between July and October 2020; of the 261 people with dementia and 206 carers recruited into DETERMIND, 114 carers and 93 people with dementia were interviewed. Seventy‐four (80%) of the people with dementia and 107 (94%) of the carers completed the questionnaires over the phone with a researcher; the remainder completed a hard copy and sent it back to the research team. The characteristics of carers and people with dementia are presented in Table 1. For carers, average age at baseline was 66 years; 76 (67%) were co‐resident with the person with dementia. Average age at baseline of the people with dementia was 80; ≈90% of the carers and people with dementia were of White British ethnicity. About three quarters of people with dementia scored > 19 in MMSE, suggesting mild or minimal cognitive impairment. The diagnosis of dementia (62% AD, 11% vascular, and 4% Lewy body) was received about 3.4 months (standard deviation [SD] = 3.27) before the baseline. Carers spent on average 5.9 hours (SD = 6.78) a day caring for the person with dementia at baseline. Table 2 presents distributions in whole sample of people with dementia that included 52 individuals who did not have a carer or where the carer did not participate in the study (and were therefore not included in Table 1). There were no statistically significant differences in participants’ characteristics between the subsample completing the C19 interview and the baseline sample (*P *> .05 for Chi‐square for categorical variables and *t*‐test for means). The mean number of memory clinic contacts reported in the North‐East was maintained (2.2 [SD = 1.52] at baseline, 2.5 [SD = 3.98] at follow‐up), while in South London and Sussex there was marked decline (baseline South London 1.4 [SD = 1.18], follow‐up 0.04 [SD = 0.19]; baseline Sussex 1.3 [SD = 1.17], follow‐up 0.14 [SD = 0.54]; Table 3).

**TABLE 1:** Distribution of carer sociodemographic variables at the DETERMIND baseline and DETERMIND‐C19 follow‐up

	*n*	Baseline %/mean (*sd*)	*n*	C19 interview %/mean (*sd*)
Location	206		114	
Sussex		47		45
North‐East		26		30
London		27		25
Age at baseline, carer	206	66.5 (13.86)	114	66.1 (13.81)
Female, carer	206	69	114	67
White British ethnicity, carer	206	91	114	90
Marital status, carer	205		114	
Married		82		82
Widowed		3		4
Separated/divorced		7		4
Single		8		11
Education, carer	198		108	
No qualification		12		7
Lower secondary school (O‐level/GCSE)		26		24
Upper secondary school (A/AS level)/vocational degree (NVQ 1‐4 levels)		33		34
Higher education degree		29		34
Occupational class, carer	183		101	
Professional		43		44
Intermediate		33		36
Routine		25		21
Work status, carer				
Working	205	28	113	24
Volunteering	205	12	113	16
Unemployed	205	5	113	6
Retired	205	61	113	65
Fulltime carer	205	16	113	18
Homemaker	205	14	113	13
Home owner, carer	206	79	114	80
Rural, carer (vs. Urban)	203	15	112	13
IMD, carer (higher=less deprived)	202	6.6 (2.79)	112	6.4 (2.85)
Coresident with person with dementia	206	67	114	67
Age at baseline, person with dementia	204	80.3 (8.25)	114	79.8 (8.85)
Female, person with dementia	206	55	114	58
White British ethnicity, person with dementia	206	92	113	92
Marital status, person with dementia	206		114	
Married		60		64
Widowed		30		26
Separated/divorced		8		8
Single		2		2
Education, person with dementia	186		104	
No qualification		32		36
Lower secondary school (O‐level/GCSE)		29		25
Upper secondary school (A/AS level)/ Vocational degree (NVQ 1‐4 levels)		23		21
Higher education degree		16		18
Occupational class, person with dementia	199		108	
Professional		37		35
Intermediate		27		27
Routine		36		38
Social benefit, person with dementia				
Pension credit	188	24	103	20
Attendance allowance	204	33	112	35
Disability living allowance	204	6	112	7
Home owner, person with dementia	206	72	114	74
Rural, person with dementia (vs. Urban)	206	11	114	11
IMD, person with dementia (higher=less deprived)	206	6.3 (2.90)	114	6.2 (2.92)
MMSE score baseline, person with dementia	204	22.0 (5.22)	113	22.3 (5.50)
MMSE score cut‐offs baseline	204			
None or minimal 26‐30		30		35
Mild 20‐25		42		39
Moderate 10‐19		25		22
Severe 0‐9		2		4
Dementia type	202		110	
Alzheimer's disease		61		59
Vascular		11		15
Lewy body		4		5
Mixed		17		14
Other		6		6
N of months since the diagnosis of dementia at baseline	176	3.7 (2.67)	95	3.6 (1.81)
N of months between baseline and C19 interview	–	–	113	8.2 (1.79)
N of hours/day caring for person with dementia at baseline	189	5.9 (6.78)	104	6.4 (7.02)

Abbreviations: IMD, Index of Multiple Deprivation; MMSE, Mini‐Mental State Examination.

**TABLE 2:** Distributions of person with dementia sociodemographic variables at the DETERMIND baseline and DETERMIND‐C19 follow‐u**p**

	n	Baseline all %/mean (*sd*)	n	C19 interview %/mean (sd)
Location	261		140	
Sussex		47		46
North‐East		25		28
London		28		26
Carer/person with dementia interview	258		137	
person with dementia only		20		17
Non‐coresident carer and person with dementia		26		28
Coresident carer and person with dementia		53		55
Age at baseline, person with dementia	252	80.2 (8.14)	137	79.5 (8.56)
Female, person with dementia	254	56	137	58
White British ethnicity, person with dementia	255	90	137	91
Marital status, person with dementia	254		137	
Married		52		58
Widowed		33		28
Separated/divorced		9		10
Single		5		3
Education, person with dementia	237		126	
No qualification		31		33
Lower secondary school (O‐level/GCSE)		27		24
Upper secondary school (A/AS level)/ Vocational degree (NVQ 1‐4 levels)		24		23
Higher education degree		18		20
Occupational class, person with dementia	244		129	
Professional		40		38
Intermediate		26		28
Routine		34		34
Social benefit, person with dementia				
Pension credit	229	24	123	21
Attendance allowance	249	28	133	31
Disability living allowance	249	6	133	7
Home owner, person with dementia	254	73	137	74
Rural, person with dementia (vs. Urban)	255	12	137	13
IMD, person with dementia (higher=less deprived)	255	6.3 (2.83)	137	6.2 (2.88)
MMSE score baseline, person with dementia	259	22.5 (5.09)	139	22.9 (5.33)
MMSE score cut‐offs baseline	259		139	
None or minimal 26‐30		34		40
Mild 20‐25		41		38
Moderate 10‐19		23		19
Severe 0‐9		2		3
Dementia type	247		133	
Alzheimer's disease		62		61
Vascular		11		15
Lewy body		4		4
Mixed		15		14
Other		8		7
N of months since the diagnosis of dementia at baseline	212	3.8 (2.71)	74	3.8 (1.79)
N of months between baseline and C19 interview	–	–	140	8.2 (1.86)

Abbreviations: IMD, Index of Multiple Deprivation; MMSE, Mini‐Mental State Examination.

**TABLE 3:** Carer‐reported number of memory clinic contacts three months before the baseline (DETERMIND) and during the pandemic (DETERMIND‐C19) by person with dementia study site

	All participants				Those present in both waves				
	*n*	Baseline mean (*sd*)	*n*	C19 mean (*sd*)	*n*	Baseline mean (*sd*)	*n*	C19 mean (*sd*)	Difference^a^
North‐East	51	2.2 (1.52)	35	2.5 (3.98)	33	2.1 (1.55)	33	2.5 (4.08)	*ns*
Sussex	93	1.3 (1.17)	49	0.1 (0.54)	46	1.3 (1.20)	46	0.2 (0.56)	***
London	53	1.4 (1.18)	27	0.0 (0.19)	26	1.0 (1.00)	26	0.0 (0.20)	***
Total	197	1.6 (1.32)	111	0.9 (2.50)	105	1.5 (1.34)	105	0.9 (2.56)	*
Difference^b^		***		***		**		***	

^a^ Paired t‐test for those present in both waves.

^b^ ANOVA between the study locations.

*ns *= non‐significant.

* *P* < .05;

** *P* < .01;

*** *P* < .001.

In terms of performance, C‐DEMQOL showed good internal consistency (baseline Cronbach's alpha 0.92, also 0.92 in DETERMIND‐C19) as did DEMQOL and DEMQOL‐Proxy (baseline Cronbach's alpha for DEMQOL 0.91 and 0.90 at follow‐up; with the respective values for DEMQOL‐Proxy 0.91 and 0.91). Table 4 shows distributions of QoL in the two interviews. Carer QoL dropped 3 points from an average 100 to 97 on the C‐DEMQOL. There was little overall change in the QoL of the people with dementia either self‐reported (DEMQOL) or reported by the carer (DEMQOL‐Proxy). Characteristics of participants in the DETERMIND‐C19 interview were similar to those assessed at DETERMIND baseline (Tables 1 and 2). In terms of interpreting these changes in QoL scores, there is no established minimal important difference (MID) for C‐DEMQOL, but for the DEMQOL system more generally MID statistics ranged between 2 and 6 points.^37^

**TABLE 4:** Distribution of quality of life total scores at baseline (DETERMIND) and DETERMIND‐C19 interviews

	All participants				Those present in both waves			
	*n*	Baseline mean (*sd*)	*n*	C19 mean (*sd*)	*n*	Baseline mean (*sd*)	*n*	C19 mean (*sd*)
C‐DEMQQL carer	172	100.5 (16.75)	101	97.4 (17.21)	85	99.5 (16.41)	85	97.1 (16.60)
DEMQOL person with dementia	245	85.8 (10.01)	91	86.7 (10.12)	88	84.7 (10.54)	88	86.8 (10.12)
DEMQOL‐Proxy person with dementia	205	88.9 (14.64)	110	88.4 (15.47)	106	86.6 (14.77)	106	88.0 (15.43)

### 3.2 Change in QoL between baseline and DETERMIND‐C19 interview

In the unadjusted model for carers, the initial level (intercept) of QoL (C‐DEMQOL) was 100.4 (standard error [SE] = 1.3, *P* < .001). The slope estimate was –2.7 (SE = 1.2, *P* = .027) indicating a decline of nearly 3 points between the baseline and DETERMIND‐C19 interviews. The residual variances for intercept and slope were 283.3 (SE = 26.7, *P* < .001) and 141.4 (SE = 25.8, *P* < .001) suggesting statistically significant individual differences in the initial levels and slopes between the carers. The model for the carers showed a good fit: χ2 = 48.52, degrees of freedom (df) = 1, CFI = 1.00, RMSEA < 0.001, SRMR < 0.001.

In the unadjusted model for the people with dementia, the initial level (intercept) of QoL was 88.9 (SE = 1.0, *P* < .001) for carer‐rated (DEMQOL‐Proxy) and 90.5 (SE = 0.9, *P* < .001) for self‐rated (DEMQOL) QoL. The slope estimates in both suggested no change in carer‐rated QoL between baseline and follow‐up (0.7, SE = 1.1, *P* = .550 for DEMQOL‐Proxy) and slight increase in self‐rated QoL (2.2, SE = 1.0, *P* = .038 for DEMQOL). There were individual differences in initial levels with some increasing and some decreasing over time. Both models for people with dementia showed a good fit: χ^2 ^= 12.66, df = 1, CFI = 1.00, RMSEA < 0.001, SRMR < 0.001 for DEMQOL, and χ^2 ^= 53.99, df = 1, CFI = 1.00, RMSEA < 0.001, SRMR < 0.001 for DEMQOL‐Proxy.

### 3.3 Association of characteristics of the carer and person with dementia with change in QoL

There was substantial individual variance around the change in carer QoL. To investigate if participants’ characteristics could explain these changes, linear regressions using the intercept and slope parameters as outcomes were carried out as part of latent growth curve estimation. First, bivariate models with one predictor at a time were fitted. Then those predictors that were associated with either the intercept or slope were included in a multivariable model. All models fitted the data well (CFI > 0.95, RMSEA < 0.05, SRMR < 0.05).

**FIGURE 1:**
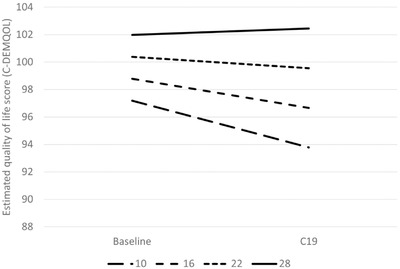
Estimated quality of life among carers (C‐DEMQOL total score) at baseline and C19 interview by cognitive impairment (Mini‐Mental State Examination [MMSE] score, higher score = less impaired) at the baseline (n = 206). To illustrate the shape of the interaction the examples of slopes for the MMSE scores 10, 16, 22 (approximate sample mean), and 28 were calculated from the latent growth curve multivariable model (Table 5). Adjusted for location, age, and sex of the person with dementia and carer; marital status, educational level, and index of multiple deprivation (carer); whether receives attendance allowance (person with dementia); number of hours caring for person with dementia/day; and number of months between baseline and C19 interview

**FIGURE 2:**
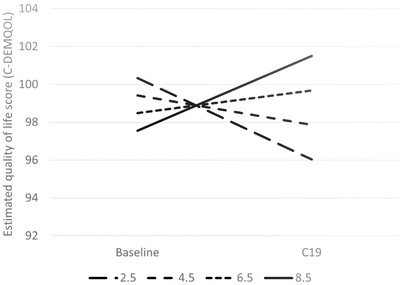
Estimated quality of life among carers (C‐DEMQOL total score) at baseline and C19 interview by area deprivation (Index of Multiple Deprivation [IMD] score, higher score = less deprived) at the baseline (n = 206). To illustrate the shape of the interaction the examples of slopes for the IMD scores 2.5, 4.5, 6.5 (approximate sample mean), and 8.5 were calculated from the latent growth curve multivariable model (Table 5). Adjusted for location, age, and sex of the person with dementia and carer; marital status and educational level (carer); whether receives Attendance Allowance and Mini‐Mental State Examination (MMSE) score; number of hours caring for person with dementia/day; and number of months between baseline and C19 interview

**FIGURE 3:**
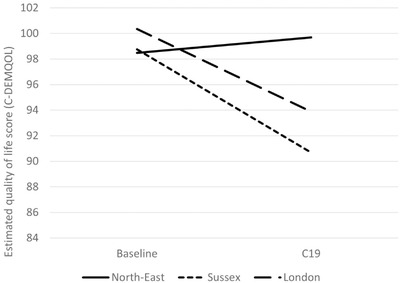
Estimated quality of life among carers (C‐DEMQOL total score) by site at baseline and C19 interview (n = 206) from the latent growth curve multivariable model (Table 5). Adjusted for age and sex of the person with dementia and carer; marital status, educational level, and index of multiple deprivation (carer); whether receives attendance allowance and Mini‐Mental State Examination (MMSE) score; number of hours caring for person with dementia/da; and number of months between baseline and C19 interview

Higher levels of cognitive impairment (lower MMSE scores) of the person with dementia were associated with greater decline in carer QoL (Table 5 and Figure 1). Similarly, higher area deprivation (lower IMD decile) was associated with greater decline in carer QoL (Table 5 and Figure 2). The results showed decline in QoL in carers of people with dementia living in Sussex and London between the baseline and DETERMIND‐C19 interviews, while the North‐East carers showed no change (Table 5 and Figure 3). After adjusting for the number of memory clinic contacts, the differences in the slopes of carer quality of life among the three study locations diminished. This suggests that each has an effect on carer QoL. Receiving Attendance Allowance, longer time since dementia diagnosis, and lower MMSE score were associated with lower carer QoL. Some factors were significant only in the bivariate analysis (carers who were older, homeowners, and had lower education showed higher QoL), and became non‐significant in the multivariable model (Table 5).

**TABLE 5:** Associations of level (intercept) and change (slope) of carer quality of life (C‐DEMQOL) with baseline characteristics of the carer and person with dementia (n = 206)

	Bivariate models for quality of life		Multivariable model for quality of life		Multivariable model for quality of life + adjusted for memory clinic contacts	
Person with dementia and carer characteristics	Intercept estimate (SE)	Slope estimate (SE)	Intercept estimate (SE)	Slope estimate (SE)	Intercept estimate (SE)	Slope estimate (SE)
Location (ref. North‐East)						
Sussex	1.8 (3.17)	−5.5 (2.68)*	0.3 (3.39)	−8.1 (2.53)**	0.6 (3.45)	−5.7 (3.02)
London	4.4 (3.69)	−6.9 (3.70)	1.9 (3.62)	−6.5 (3.40)	2.1 (3.72)	−3.5 (3.78)
Age at baseline, person with dementia	0.0 (0.14)	0.2 (0.11)	0.2 (0.18)	0.0 (0.13)	0.2 (0.18)	0.0 (0.14)
Female, person with dementia	−0.5 (2.51)	4.0 (2.40)	0.7 (3.15)	0.4 (2.62)	1.0 (3.17)	−1.5 (2.80)
White British ethnicity, person with dementia	3.9 (6.12)	−5.9 (5.19)	–	–	–	–
Married, person with dementia	0.5 (2.63)	−3.2 (2.45)	–	–	–	–
Education, person with dementia (ref = no qualification)						
Lower secondary school (O‐level/GCSE)	3.2 (3.35)	−4.8 (3.31)	–	–	–	–
Upper secondary school (A/AS level)/Vocational degree (NVQ 1‐4 levels)	1.2 (3.49)	−4.6 (3.64)	–	–	–	–
Higher education degree	−1.7 (4.19)	−0.3 (4.40)	–	–	–	–
Social benefit, person with dementia						
Pension credit	2.0 (2.88)	−2.3 (3.42)	–	–	–	–
Attendance allowance	−7.7 (2.63)**	−0.2 (2.73)	−6.2 (2.77)*	−3.8 (2.23)	−6.4 (2.78)*	−3.7 (2.43)
Disability living allowance	0.5 (4.69)	−0.2 (3.52)	–	–	–	–
Occupational class, person with dementia (ref = routine)						
Intermediate	2.4 (3.25)	−0.2 (3.02)	–	–	–	–
Professional	2.5 (2.97)	−1.5 (3.00)	–	–	–	–
Home owner, person with dementia	3.4 (2.77)	−1.5 (2.82)	–	–	–	–
Age at baseline, carer	0.2 (0.09)*	−0.1 (0.10)	0.0 (0.11)	−0.1 (0.12)	0.0 (0.11)	−0.1 (0.14)
Female, carer	−2.4 (2.68)	1.8 (2.61)	−3.1 (3.00)	0.3 (2.56)	−2.9 (3.00)	0.5 (2.41)
White British ethnicity, carer	4.9 (5.55)	−6.4 (5.19)	–	–	–	–
Married, carer	6.2 (3.56)	−5.5 (2.58)	5.0 (3.68)	−3.4 (3.95)	5.0 (3.64)	−4.6 (4.30)
Education, carer (ref=no qualification)						
Lower secondary school (O‐level/GCSE)	−5.8 (4.57)	−0.2 (4.70)	−1.2 (4.15)	−4.6 (5.23)	−1.0 (4.17)	−5.8 (5.06)
Upper secondary school (A/AS level)/Vocational degree (NVQ 1‐4 levels)	−9.3 (4.37)*	6.1 (4.63)	−4.6 (3.92)	3.9 (5.28)	−4.5 (3.94)	3.3 (5.19)
Higher education degree	−9.6 (4.54)*	5.8 (4.38)	−7.3 (4.12)	3.9 (5.28)	−7.3 (4.11)	2.2 (5.44)
Occupational class, carer (ref = routine)						
Intermediate	0.5 (3.75)	−3.3 (4.74)	–	–	–	–
Professional	0.4 (3.67)	1.3 (4.71)	–	–	–	–
Home owner, carer	6.6 (2.81)*	−0.3 (3.20)	0.7 (3.14)	2.5 (3.45)	0.7 (3.14)	2.6 (3.63)
Work status, carer						
Working	−0.4 (5.20)	−0.8 (5.54)	–	–	–	–
Volunteering	−2.4 (3.45)	−1.1 (3.08)	–	–	–	–
Unemployed	−6.8 (6.04)	1.3 (5.60)	–	–	–	–
Retired	5.2 (4.83)	−3.3 (5.04)	–	–	–	–
Fulltime carer	−5.4 (3.43)	0.1 (3.66)	–	–	–	–
Homemaker	0.1 (4.24)	−1.9 (5.35)	–	–	–	–
Rural, carer (ref = Urban)	3.0 (2.72)	−4.0 (3.28)	–	–	–	–
IMD, carer (higher=less deprived)	−0.4 (0.49)	0.7 (0.30)*	−0.6 (0.46)	1.4 (0.44)**	−0.6 (0.47)	1.5 (0.41)***
Carer coresident with person with dementia	1.1 (2.65)	−4.9 (2.69)	–	–	–	–
MMSE score baseline, person with dementia	0.8 (0.22)***	−0.4 (0.23)	0.5 (0.23)*	−0.5 (0.21)*	0.5 (0.23)*	−0.5 (0.20)*
Dementia type (ref=Alzheimer's disease)						
Vascular	−2.9 (4.05)	−2.1 (2.81)	–	–	–	–
Lewy body	1.6 (4.15)	0.5 (4.25)	–	–	–	–
Mixed	−1.5 (3.44)	1.1 (3.58)	–	–	–	–
Other	1.8 (5.39)	3.3 (4.30)				
N of hours/day caring for person with dementia at baseline	−0.6 (0.19)**	−0.2 (0.15)	−0.4 (0.19)*	−0.2 (0.15)	−0.4 (0.19)*	−0.2 (0.15)
N of months since the diagnosis of dementia at baseline	−0.8 (0.45)	0.2 (0.94)	−0.9 (0.40)*	1.8 (0.84)*	−0.9 (0.40)*	1.9 (0.76)*
N of months between baseline and C19 interview	−0.5 (0.73)	0.01 (0.63)	−0.7 (0.76)	−0.1 (0.63)	−0.7 (0.76)	−0.3 (0.61)
*Time‐varying predictors*	*Baseline*	*C19*			*Baseline*	*C19*
N of memory clinic contacts baseline	−0.9 (0.79)	–	–	–	0.5 (0.83)	–
N of memory clinic contacts C19 interview	–	0.6 (0.34)	–	–	–	0.8 (0.38)*

Abbreviations: IMD, Index of Multiple Deprivation; MMSE, Mini‐Mental State Examination.

* *P* < .05;

** *P* < .01;

*** *P* < .001. Unstandardized estimates and standard errors (SE) shown.

The bivariate and multivariable analyses revealed few relationships between the person with dementia's change in QoL and their characteristics (Tables S1 and S2 in supporting information). None of the characteristics were associated with the change in DEMQOL‐Proxy in the fully adjusted model (Table S1). Longer time since dementia diagnosis, receiving Attendance Allowance, and carer's higher educational level were associated with lower DEMQOL‐Proxy scores in the multivariable model (Table S1). Longer time since dementia diagnosis was associated with increased DEMQOL‐Proxy scores while carer co‐residence was associated with higher and receiving Disability Allowance with lower QoL in the bivariate analysis only. Non‐white ethnicity and receiving Disability Living Allowance were associated with decrease in DEMQOL in the multivariable model (Table S2). Homeownership, and shorter time since dementia diagnosis and time between the baseline and C19 interviews were associated with higher baseline DEMQOL scores in the multivariable model.

## 4 DISCUSSION

This is the first study exploring the impact of COVID‐19 and governmental restrictions on the QoL of people newly diagnosed with dementia and their carers. Our results suggest there has been a maintenance of QoL of people with dementia during the pandemic, but that this may have come at the expense of the QoL of their family carers, which declined over this time. This decrease in carer QoL was largest in those supporting people with more severe dementia, and those in more deprived areas. While causation cannot be inferred from the study design, these results suggest the possibility that these negative effects might be mitigated by continued post‐diagnostic care because those carers for whom memory services continued to support people with dementia had better outcomes than those for whom such support services were largely withdrawn.

Our finding that the QoL of people with dementia did not decrease was unexpected. Given that the DEMQOL system is psychometrically robust and was developed specifically for the measurement of QoL in dementia, it suggests that, with the help of family carers, people with dementia were able to maintain their QoL despite the sudden disruption imposed by COVID‐19. The resilience of people with dementia is often underestimated.^38^ However, these data were collected between July and October 2020, during the UK summer and a partial lifting of the restrictions. It is possible that participants had come to terms with the initial shock of the pandemic and had begun to develop mechanisms to counter any potentially adverse impacts. Our findings fit with reports that QoL may have been maintained^24^ or even have increased for some with dementia.^22^ It is possible that, as in other research,^17^ having been recently diagnosed with dementia, our participants benefitted from the lockdown bubble and shrinking of their worlds as a means to come to terms with their dementia diagnosis in a safe environment. We will explore this in the qualitative component of DETERMIND‐C19. Experiences are likely to have been very different for those with dementia in care homes.

Our findings for carers are of concern and in line with reports of an increase in depressive symptoms and anxiety in carers generally^20^, ^21^ and in depression among carers of people with dementia specifically as a consequence of COVID‐19 and the public health restrictions.^22^ During the pandemic, the QoL of the DETERMIND carers decreased. This was measured using an instrument specifically developed for use with family carers of people with dementia that quantifies how caring as a whole, with its positive and negative aspects, has affected the carer. Those carers who were supporting people with higher levels of cognitive impairment experienced a greater decline in QoL in the pandemic. Difficulties accessing formal health and social care services, together with limits on community activities and restricted informal social networks, will have placed a high level of strain on these carers. They may also have opted against (re)employing paid home care or less formal paid and unpaid arrangements for fear that introducing others into their household would increase their risk of infection; thereby resulting in a need for them to provide additional care at a cost to their own well‐being.^39^

Those carers living in areas of high deprivation were most affected. This demonstrates that there are social determinants of outcomes for carers in addition to the influence of characteristics of those for whom they care. The variation among the three recruitment sites is striking and offers the hope that these harms to carers might be prevented or reversed by appropriate service provision. Part of the variation among sites was due to differences in deprivation, but more was associated with service delivery. We found that support from memory services in the North‐East had continued during the pandemic while those in South London and Sussex decreased. While the design of our study does not allow for inference of causality, our modeling suggests that continued contact from memory services during the pandemic was protective of the QoL of carers. This association seems independent of the effects of deprivation and severity of dementia. Our data provide support for the potential value of post‐diagnostic care in supporting carers of people with dementia and preventing these harms. Pre‐pandemic, diagnostic, and post‐diagnostic services were a “postcode lottery”^40^, ^41^ and this is only likely to have widened in the last year. Post‐diagnostic care is key to enabling people to live well with dementia and to preventing harms. The majority of post‐diagnostic care is provided by primary and community care teams with memory clinics and secondary care services providing expert specialist advice when needed. However, what should be provided and by whom is underspecified in commissioning terms in the UK and there is marked variability with very little provided to many.^41^ There is a need to develop better post‐diagnostic care in dementia and the data presented here suggest how valuable such services may be, even in the most challenging of circumstances.

There are important limitations to this study. The first is inherent in the DETERMIND cohort that was designed to investigate care inequalities and so includes variation in characteristics that might be associated with variations in care such as ethnicity, social class, and region. It is therefore not a representative sample of the UK population of people with dementia.^25^ However, this variation in deprivation may have helped us to identify the associations found by increasing the statistical power for subgroup analyses. Second, we were unable to recruit all eligible participants into DETERMIND‐C19. This non‐response, including people with dementia who lacked capacity at baseline, may have caused bias in our results, but it is positive that DETERMIND‐C19 subsample was similar to the baseline DETERMIND population in sociodemographic characteristics. It may have been more likely to include those who engaged with a telephone questionnaire and so we may have missed data from participants who found the pandemic particularly challenging. We will attempt to pick this group up in further waves of DETERMIND and re‐engage with them to explore retrospectively their experiences during the pandemic. Third, we focussed on QoL, which is a broad measure of overall well‐being and did not investigate more subtle changes in psychological function, or clinical depression and anxiety. However, in a multifaceted challenge such as the pandemic and in a population as heterogeneous as those with dementia, it is particularly important to look at overall effects. In the absence of MID statistics for C‐DEMQOL, the clinical rather than the statistical significance of our findings are subject to question, but the direction of change is clear from our study. Fourth, the design of the project and the nature of the pandemic does not allow us to directly attribute the changes in carer QoL to the pandemic rather than the normal process of adjustment to caring for someone with dementia after diagnosis. Fifth, the administration of the follow‐up questionnaires coincided with a time of eased restrictions in the UK, further limiting the ability to make causal attributions of the current results to effects of the COVID‐19 pandemic; we may have obtained different results had we interviewed during the height of the lockdown. Sixth, in our mixed group of diagnoses, the MMSE has limitations as measure of cognitive decline for non‐AD diagnoses, as it might underestimate cognitive function, which could affect the relationship in the data with cognition. Finally, our data do not cover care homes where people with dementia and carers have suffered greatly during the pandemic. In terms of strengths, our study is the first to examine change in QoL using empirical data collected prior to the pandemic and repeat measurement during it, as opposed to relying on cross‐sectional or subjective retrospective ratings. The longitudinal design of DETERMIND also means that we can continue to monitor participant QoL over the years to provide insights into the long‐term impact of the pandemic.

## 5 CONCLUSIONS

Our data show that the major QoL impacts of the pandemic have been on family carers of people with dementia rather than the people with dementia. The evidence of resilience of people with dementia in QoL terms, at least in the early months of the pandemic, is encouraging and it is important that future research seek to examine positive outcomes, as well as the negative, and elicit the individual and societal facilitators that supported positive QoL. With the limitations inherent in the design of this study, these data provide support for the positive value of post‐diagnostic care, much of which has been closed by the pandemic or is only working virtually or with a much‐reduced service.^42^ Particular attention is needed to address these inequities for those caring for people with more severe dementia and those living in more deprived areas. Our findings should encourage primary and secondary care services providing memory assessment services and post‐diagnostic care to re‐open them and maintain them as a priority. This demonstration of value should also be of use to service planners in developing and commissioning good quality post‐diagnostic dementia care, supporting and training primary care teams to provide generalist support while working with specialists in more complex cases.

## CONFLICTS OF INTEREST

This project was supported by UK Research and Innovation (UKRI) through the Economic and Social Research Council (ESRC) by a research grant which supported the salaries of the research workers employed on this project (AG, AK, EM, EN, JR, AR, ES, BS) and travel expenses. The project used data from the DETERMIND project funded by UKRI/ESRC and the National Institute of Health and Social Research (NIHR) that supported the participation of all authors. Both grants were paid to institutions, not personally. The following are in addition to the above and are outside the submitted work. SB reports ESRC, UKRI, and NIHR institutional grant funding and personal fees and non‐financial support from medicolegal reports, Lilly, personal fees from Axovant, personal fees from Lundbeck, personal fees from Nutricia, and honoraria from the Hamad Medical Service and for lectures and talks. He is a Trustee of the Alzheimer's Society, Editor in Chief of the *International Journal of Geriatric Psychiatry* (personal honorarium) and a Non‐Executive Director of Somerset Partnership NHS Foundation Trust. BH reports European Union (EU) ERASMAS institutional grant funding. SR reports payment from Trinity College Dublin to her institution to attend meetings. RW reports institutional grant funding ESRC, UKRI, NIHR and its Schools for Social Care Research (SSCR) and Primary Care Research (SPCR), UKSPINE, MSD, Alzheimer's Society, Greek Government, and the EU; the Brookdale Institute funded travel and expenses for attending an international advisory board meeting. EN reports employment by the NHS as a researcher. KB reports institutional research grants including salary funding from NIHR SSCR, NIHR RfPB, NIHR HS&RD, and ESRC. She reports several leadership and advisory roles in dementia and social care organizations, all of which are unpaid. EB has institutional grant funding from NIHR SSCR, NIHR, ESRC, and Abbeyfields; she holds non‐paid advisory roles and is the NIHR SSCR Deputy Director, which pays part of her salary to the University of York, her employer. CB reports institutionally paid grants from: Alzheimer's Society, Addenbrooke's Charitable Trust, ESRC, NIHR ARC, EU, Canadian Institute of Health Research, Alzheimer's Research UK (ARUK), NIHR, National Institute on Aging/National Institutes of Health, Innovative Medicines Initiative (EPAD) Innovative Medicines Initiative Joint Undertaking, MRC, AHRC‐UKRI GCRF, the Gillings Family Foundation, and NIHR HTA. She has received institutional and personal reimbursement for travel/accommodation/subsistence expenses to attend meetings/conferences as speaker. She is a member of: NIA HRS Data Monitoring Committee with honoraria paid to employing institution (University of Cambridge) and travel and subsistence expenses reimbursed; AXA Research Fund Scientific Board (honoraria paid to employer); DBT/Wellcome Trust India Alliance Fellowship Selection Committee (honoraria paid to self, travel & subsistence expenses reimbursed), Chair of the Canadian Longitudinal Study on Aging Scientific Advisory Board (travel/subsistence expenses reimbursed); Co‐Chair of the Alzheimer's Society Research Strategy Council (travel/subsistence expenses reimbursed); Chair of the BRAIN & HEADING International Oversight Committee (travel/subsistence expenses reimbursed); member of The Irish Longitudinal Study on Aging (TILDA) Scientific Advisory Board Travel (subsistence expenses reimbursed); CUHK Project Advisory Board; University of Sheffield Health Lifespan Institute Advisory Board; ATHLOS Advisory Board (travel/subsistence expenses reimbursed); Barcelona Brain Health Initiative Scientific Advisory Board (travel/subsistence expenses reimbursed); DZNE International Scientific Review Panel (travel/subsistence expenses reimbursed); Scientific Advisory Board for UKPRP Air pollution and cognitive health consortium; and InSPIRE. She is: Chair, Faculty of Public Health Academic & Research Committee (travel/subsistence expenses reimbursed); Trustee, Faculty of Public Health Board (travel/subsistence expenses reimbursed); Chair Royal College of Physicians Advisory Group on Health Inequalities (travel/subsistence expenses reimbursed); Chair, Public Health England—University of Cambridge Academic Liaison Committee meeting; and Co‐Chair, East of England Public Health England Research and Evaluation Hub. NB reports institutional grants from NIHR HS&DR and NIHR SSCR. She receives royalties from Routledge from revenue of publications by Melanie Klein paid personally. MD has the following unpaid positions: Carers UK (Charity Trustee); The Centre for Ageing Better (Charity Trustee); Crossroads Care Richmond & Kingston (Charity Trustee); The Friends of Queen Mary's Hospital Roehampton (Charity Trustee). JD reports support from ESRC, NIHR, and UKRI; and has grants paid institutionally from the Alzheimer's Society and NIHR. KG has institutionally paid grant support from HIHR SSCR. MK has grants paid institutionally from: ARUK, Alzheimer's Society, Australian Research Council, ESRC, Health Foundation, Medical Research Council (MRC); NIHR, NIHR SSCR, Department of Health and Social Care for England (DHSC), NIHR RfPB, Open Society Foundation, UKRI, ESRC and RCN Foundation. He receives a salary from the London School of Economics and has received payment for teaching from the University of Hong Kong and the Civil Service College Singapore. RP reports support paid institutional support to attend conferences from ESRC/NIHR. RS reports institutional research funds from Janssen, a supported PhD studentship from Takeda, for supervision of a GSK employee's PhD. He has received personal royalties from Oxford University Press for two co‐edited textbooks. AT reports institutional grants from: Alzheimer's Society, and ARUKI. BHu, AG, AK, EM, EN, JR, AR, ES, BS, PH, EM, RP, LR, and JR make no other disclosures.

## AUTHOR CONTRIBUTIONS

Sube Banerjee was the chief investigator for the study and designed and managed the study with input from the group. Sanna Read carried out the statistical analyses. All authors had access to data and participated in data interpretation. Ben Hicks, Jacob Reichental, Anomita Karim, Alice Russell, Eva Tipping, Evelyn Martin, Eleanor Nuzum, Amanda Grahamslaw, Bryony Storey, and Elaine Siddle collected the data and tested the data collection systems. Sanna Read, Ben Hicks, and Bo Hu have verified the underlying data. Ben Hicks, Sanna Read, Bo Hu, Raphael Wittenberg, and Sube Banerjee drafted the first and Sube Banerjee subsequent versions of this paper with input and revisions by all authors, who reviewed and approved the final submitted paper.

## DATA AVAILABILTY STATEMENT

Deidentified participant data will be available with investigator support from 9 months after publication of the last DETERMIND‐C19 paper via sube.banerjee@plymouth.ac.uk by researchers whose proposed use of the data has been approved by the DETERMIND Programme Management Board for analyses that have been approved. The study protocol will be available as a supporting document.

## Supporting information

Supporting information
