# Supplementary material for: A cohort study of the impact of COVID‐19 on the quality of life of people newly diagnosed with dementia and their family carers
Source: Alzheimers Dement (N Y). 2022 May 2;8(1):e12236. doi: 10.1002/trc2.12236 (PMC9060551; doi:10.1002/trc2.12236)
Supplement: Supplementary file 1 — Supporting information [file TRC2-8-0-s001.docx]

***Supplementary Table 1: Bivariate associations of level (intercept) and change (slope) of the DEMQOL-Proxy of the person with dementia with the baseline characteristics of the carer and the person with dementia (n = 206).***

|  | **Bivariate models for quality of life** | | **Multivariable model for quality of life** | |
| --- | --- | --- | --- | --- |
| **Person with dementia and carer characteristics** | **Intercept**  **Unstandardized estimate (SE)** | **Slope**  **Unstandardized estimate (SE)** | **Intercept**  **estimate (SE)** | **Slope**  **estimate (SE)** |
| Location (ref. North-East) |  |  |  |  |
| Sussex | -0.6 (2.45) | -0.9 (2.81) | - | - |
| London | 1.7 (2.99) | -4.7 (3.30) | - | - |
| Age at baseline, person with dementia | -0.1 (0.14) | 0.3 (0.15) | 0.1 (0.18) | 0.2 (0.14) |
| Female, person with dementia | -1.8 (2.04) | 1.7 (2.39) | 0.6 (3.34) | 3.3 (3.11) |
| White British ethnicity, person with dementia | -3.7 (3.90) | 5.1 (5.00) | - | - |
| Married, person with dementia | 0.9 (2.07) | -0.9 (2.49) | - | - |
| Education, person with dementia (ref = no qualification) |  |  |  |  |
| Lower secondary school (0-level/GCSE) | 1.1 (2.83) | -1.1 (3.07) | - | - |
| Upper secondary school (A/AS level)/ Vocational degree (NVQ 1-4 levels) | -2.0 (2.85) | -1.2 (3.58) | - | - |
| Higher education degree | 0.6 (3.15) | 0.6 (3.43) | - | - |
| Social benefit, person with dementia |  |  |  |  |
| Pension credit | 0.7 (1.38) | -2.2 (1.76) | - | - |
| Attendance allowance | -5.6 (2.25)* | 1.4 (2.73) | -7.3 (2.75)** | -2.1 (2.49) |
| Disability living allowance | -14.0 (4.05)** | 5.1 (4.25) | 4.6 (4.38) | 0.7 (4.63) |
| Occupational class, person with dementia (ref = routine) |  |  |  |  |
| Intermediate | -0.9(2.66) | -0.4 (2.72) | - | - |
| Professional | -1.3 (2.42) | 2.2 (3.09) | - | - |
| Home owner, person with dementia | 1.4 (2.19) | 1.6 (2.84) | - | - |
| Rural, person with dementia (vs. Urban) | -3.6 (3.15) | 3.3 (3.49) | - | - |
| IMD, person with dementia (higher=less deprived) | 0.5 (0.35) | 0.2 (0.44) | - | - |
| Age at baseline, carer | 0.1 (0.08) | -0.1 (0.08) | 0.1 (0.11) | -0.1 (0.11) |
| Female, carer | 3.4 (2.27) | -0.9 (2.41) | -3.0 (3.02) | 3.6 (3.27) |
| White British ethnicity, carer | -2.6 (3.69) | 2.9 (4.68) | - | - |
| Married, carer | -1.0 (2.63) | -1.7 (3.30) | - | - |
| Education, carer (ref=no qualification) |  |  |  |  |
| Lower secondary school (O-level/GCSE) | -9.4 (3.49)** | 11.7 (5.08)* | -2.2 (4.44) | -3.1 (5.40) |
| Upper secondary school (A/AS level)/ Vocational degree (NVQ 1-4 levels) | -9.8 (3.06)** | 8.7 (5.17) | -6.0 (4.39) | 4.4 (5.25) |
| Higher education degree | -8.3 (3.22)* | 7.1 (5.15) | -8.8 (4.42)* | 3.5 (5.33) |
| Occupational class, carer (ref = routine) |  |  |  |  |
| Intermediate | -4.2 (3.03) | 2.5 (3.76) | - | - |
| Professional | -1.1 (2.67) | -0.4 (3.81) | - | - |
| Home owner, carer | 3.1 (2.36) | 0.4 (3.27) | - | - |
| Work status, carer |  |  |  |  |
| Working | -0.04 (4.21) | -1.8 (5.58) | - | - |
| Volunteering | 1.1 (3.55) | 2.5 (3.50) | - | - |
| Unemployed | -4.6 (4.30) | -2.8 (4.85) | - | - |
| Retired | 3.2 (3. 97) | -2.2 (5.27) | - | - |
| Fulltime carer | 0.4 (2.86) | -6.3 (3.91) | - | - |
| Homemaker | -2.4 (2.76) | -2.7 (4.50) | - | - |
| Rural, carer (ref = Urban) | -1.2 (2.92) | 0.7 (3.29) | - | - |
| IMD, carer (higher=less deprived) | 0.1 (0.37) | 0.2 (0.42) | - | - |
| Carer coresident with person with dementia | 4.3 (2.18)* | -1.3 (2.59) | -4.4 (3.79) | 0.9 (3.48) |
| MMSE score baseline, person with dementia | 0.1 (0.22) | 0.1 (0.21) | - | - |
| Dementia type (ref=Alzheimer’s disease) |  |  |  |  |
| Vascular | -3.2 (3.75) | -0.1 (3.79) | - | - |
| Lewy body | -5.3 (5.82) | 4.3 (6.42) | - | - |
| Mixed | 1.9 (2.57) | -2.1 (3.11) | - | - |
| Other | 0.6 (4.33) | 0.2 (4.42) | - | - |
| N of hours/day caring for person with dementia at baseline | -0.3 (0.17) | -0.2 (0.20) | - | - |
| N of months since the diagnosis of dementia at baseline | -1.1 (0.23)** | 1.5 (0.61)* | -1.3 (0.51)* | 1.0 (0.64) |
| N of months between baseline and C19 interview | -0.65 (0.55) | -0.23 (0.71) | -0.9 (0.70) | 0.1 (0.64) |
| *Time-varying predictors* | *Baseline* | *C19* |  |  |
| N of memory clinic contacts baseline | 0.1 (0.88) | - | - | - |
| N of memory clinic contacts C19 interview | - | -0.2 (0.51) | - | - |

IMD= Index of Multiple Deprivation, MMSE=Mini-Mental State Examination. * *p* < 0.05, ** *p* < 0.01 , *** *p* < 0.001 .

***Supplementary Table 2: Bivariate associations of level (intercept) and change (slope) of the self-rated quality of life (DEMQOL) of the person with dementia with the baseline characteristics of the person with dementia (n = 261).***

|  | **Bivariate models for quality of life** | | **Multivariable model for quality of life** | |
| --- | --- | --- | --- | --- |
| **Person with dementia characteristics** | **Intercept**  **Unstandardized estimate (SE)** | **Slope**  **Unstandardized (SE)** | **Intercept**  **Unstandardized estimate (SE)** | **Slope**  **Unstandardized (SE)** |
| Location (ref. North-East) |  |  |  |  |
| Sussex | -0.3 (1.63) | -1.8 (2.00) | -0.1 (1.94) | -2.3 (2.18) |
| London | -1.1 (1.76) | -5.9 (2.86)* | -1.3 (1.80) | -4.2 (2.69) |
| Dyad type (ref = person with dementia only) |  |  |  |  |
| Non-coresident carer and person with dementia | -0.1 (2.64) | 2.2 (2.50) | - | - |
| Coresident carer and person with dementia | 2.3 (2.18) | -0.6 (2.48) | - | - |
| Age at baseline, person with dementia | -0.1 (0.08) | 0.01 (0.10) | 0.1 (0.08) | 0.1 (0.11) |
| Female, person with dementia | 0.5 (1.29) | 1.2 (1.73) | -0.4 (1.99) | 3.5 (2.38) |
| White British ethnicity, person with dementia | 1.6 (2.61) | 9.5 (4.47)* | 1.5 (2.63) | 10.0 (4.62)* |
| Married, person with dementia | -0.4 (1.30) | -2.2 (1.71) | - | - |
| Education, person with dementia (ref = no qualification) |  |  |  |  |
| Lower secondary school (O-level/GCSE) | 1.1 (1.72) | -1.3 (1.88) | - | - |
| Upper secondary school (A/AS level)/ Vocational degree (NVQ 1-4 levels) | 0.3 (1.77) | -2.4 (2.38) | - | - |
| Higher education degree | 0.8 (2.15) | -0.9 (2.57) | - | - |
| Social benefit, person with dementia |  |  |  |  |
| Pension credit | 0.8 (1.57) | -3.5 (1.91) | - | - |
| Attendance allowance | -2.3 (1.53) | -0.9 (2.44) |  |  |
| Disability living allowance | -5.2 (3.35) | -5.8 (1.85)** | -3.0 (3.15) | -9.6 (2.58)*** |
| Occupational class, person with dementia (ref = routine) |  |  |  |  |
| Intermediate | 2.4 (1.74) | -1.9 (2.13) | - | - |
| Professional | 2.8 (1.52) | -1.3 (2.26) | - | - |
| Home owner, person with dementia | 3.7 (1.54)* | -0.3 (2.08) | 3.9 (1.63)* | 0.8 (1.97) |
| Rural, person with dementia (vs. Urban) | 0.4 (1.86) | 1.3 (1.88) | - | - |
| IMD, person with dementia (higher=less deprived) | 0.3 (0.24) | 0.3 (0.28) | - | - |
| MMSE score baseline, person with dementia | 0.2 (0.14) | -0.1 (0.19) | - | - |
| Dementia type (ref=Alzheimer’s disease) |  |  |  |  |
| Vascular | -0.8 (2.15) | -1.2 (2.96) |  |  |
| Lewy body | -3.7 (3.52) | 2.3 (2.58) |  |  |
| Mixed | 0.3 (1.64) | -0.9 (2.70) |  |  |
| Other | -1.3 (1.64) | -2.1 (3.27) |  |  |
| N of months since the diagnosis of dementia at baseline | -0.5 (0.23)* | -0.8 (0.55) | -0.5 (0.23)* | -0.3 (0.54) |
| N of months between baseline and C19 interview | -0.6 (0.34) | 0.2 (0.37) | -0.7 (0.34)* | -0.0 (0.42) |
| *Time-varying predictors* | *Baseline* | *C19* |  |  |
| N of memory clinic contacts baseline | 0.1 (0.80) | - | - | - |
| N of memory clinic contacts C19 interview | - | 0.0 (0.35) | - | - |

IMD= Index of Multiple Deprivation, MMSE=Mini-Mental State Examination. * *p* < 0.05, ** *p* < 0.01 , *** *p* < 0.001.
